# Rapid Characterization of Middle-Ear Muscle Reflexes Using Swept Elicitors

**DOI:** 10.1007/s10162-026-01037-z

**Published:** 2026-03-23

**Authors:** M. Ehsan Khalili, Julia H. Roemen, Jeffery T. Lichtenhan, Shawn S. Goodman

**Affiliations:** 1 Department of Communication Sciences and Disorders, University of Iowa, Iowa City, IA USA; 2 Department of Otolaryngology – Head and Neck Surgery, University of South Florida, Tampa, FL USA; 3 Turner Scientific, LLC., Jacksonville, IL USA

**Keywords:** Acoustic reflex, Total change, Hysteresis, Speech-in-noise, Hearing thresholds, Wideband acoustics

## Abstract

**Purpose:**

We describe a novel paradigm for evoking and measuring middle ear muscle reflex (MEMR), in which a train of broadband clicks act as probes, while a broadband noise elicitor is continuously swept in both ascending and descending sound levels. A new measure, total change, incorporates both magnitude and phase to quantify MEMR in a way that promotes meaningful averaging across a wide range of sound levels and frequencies. The aims of the study were to assess the retest reliability of the new swept-elicitor MEMR paradigm, to compare results with those obtained using traditional discrete-elicitor stimuli, and to preliminarily examine correlations with speech-in-noise performance.

**Methods:**

MEMR was measured in 38 young, normal-hearing participants (24 female, 14 male) using both the novel swept paradigm and a more conventional paradigm with elicitor noises that were discretely varied in level. Key measures of MEMR dynamics were obtained from the swept elicitor paradigm, including maximum total change, onset and offset thresholds, hysteresis, and reflex delay. Intraclass correlation coefficients (ICCs) were used to assess repeatability, and robust linear regression was used to examine correlations with QuickSIN performance.

**Results:**

The swept MEMR paradigm demonstrated excellent repeatability, with ICC values exceeding 0.90 for all extracted measures. MEMR thresholds from the swept elicitor correlated moderately with speech-in-noise performance.

**Conclusions:**

Our new MEMR paradigm provides fast, repeatable measurements. Several measures of MEMR dynamics can be obtained, improving upon traditional measurement approaches. Results suggest a possible link between MEMR dynamics and speech-in-noise performance.

## Introduction

 The middle ear muscle reflex (MEMR), involving the stapedius and/or tensor tympani muscles, is a unique feedback mechanism in auditory physiology. These muscles are innervated by efferent fibers originating near the facial and trigeminal nerve nuclei [26]. The stapedius muscle is activated by moderate to loud external sounds, while the tensor tympani muscle plays a larger role in response to self-generated noises such as swallowing, mastication, or sneezing [34]. In both cases, the reflex is bilateral and can be observed in both ears. Contractions of these muscles increase middle ear stiffness, altering its resonance, which is typically located around 800–1200 Hz [36, 49]. As a result of MEMR activation, stimulus frequencies below the middle ear resonance encounter an increased impedance mismatch, reducing transmission of low frequencies into the cochlea. Conversely, frequencies in the region just above the resonance encounter a reduced impedance mismatch, slightly increasing transmission into the cochlea. Activation of the MEMR may reduce the masking of high-frequency sounds (e.g., consonants) by low-frequency background noise [25, 37]. Additionally, several studies suggest that MEMR may offer protection from noise-induced hearing loss, including exposure to intense impulsive sounds [5, 6, 43, 46]. However, the extent of these performance and protective effects will likely continue to be incompletely understood by the broader research community.

MEMR measurements are a common component of audiological diagnostic assessment, often included in test batteries for the purposes of determining consistency across tests, differentiating conductive and sensorineural hearing loss, and helping diagnose retrocochlear pathologies [18, 22]. In combination with tympanometry and behavioral audiometry, MEMR has been shown to be effective for screening and detecting conductive hearing loss and for estimating the degree of cochlear hearing loss [9, 14, 19, 21, 28, 42]. MEMR may also be a useful part of diagnosing “auditory desynchrony” [3].

Traditional methods of measuring MEMR rely on time-consuming use of elicitors at discrete sound frequencies and levels while responses are measured with a 226 Hz, fixed-level probe tone. Several studies have suggested using wideband clicks as probes, sometimes paired with wideband noise elicitors, for greater sensitivity in detecting MEMR changes and obtaining lower thresholds [13, 21]. Some of these improvements presumably arise from accounting for variations across participants by allowing the frequencies most sensitive to MEMR-induced change to dominate the analyzed responses. The use of wideband elicitors also results in lower MEMR thresholds compared to pure tones [28, 50], presumably due to broader cochlear excitation patterns and increased overall neural activation.

Experimental research studies have sought to expand the utility of the MEMR beyond its traditional use in the clinic. Studies have shown that wideband MEMR assessments meet or exceed the performance of traditional methods in evaluating MEMR [7, 28, 41, 44]. MEMR has been used for early identification of hearing loss [45, 46], enhancing our understanding of the role of the middle ear in hearing [34], and for differential diagnosis of middle ear disease [18]. Recent studies on hidden hearing loss have studied MEMR as a potential assay of synaptopathy (i.e., loss of synapses between inner hair cells and high-threshold auditory nerve fibers) that may be an origin of hidden hearing loss [46, 47, 51]; therefore, assessing the MEMR over a wide range of elicitor levels can give a more complete description of MEMR dynamics and may aid in objective assessment of speech-in-noise performance. However, to date, wideband MEMR techniques have been limited to the identification of MEMR thresholds and MEMR magnitude change, without capturing other potentially useful dynamics, such as hysteresis, reflex delay, and level growth function (LGF) slopes. It is possible that inclusion of these dynamic aspects could help advance the use of wideband MEMR assessment.

Here, we describe a rapid, 2-min MEMR paradigm which presents wideband noise elicitors sweeping over a range of increasing and decreasing sound levels, along with a train of wideband click probes. Analyzed results include LGFs, maximum reflex activation, onset and offset thresholds, hysteresis, and reflex delay. This study had two primary goals: (1) to assess the retest reliability of our new swept-elicitor MEMR paradigm and (2) to compare the results of the swept-elicitor paradigm with those obtained using similar stimuli but in a discrete presentation format. The study also examined correlations between various measures from the swept-elicitor paradigm with performance on the QuickSIN speech-in-noise (SIN) test.

## Methods

### Participants

For the main results of this study, MEMRs were measured from 30 young, normal-hearing participants (16 females; mean 20.1 years, range 18 to 30 years). Additional MEMR measurements were made from a separate cohort of eight participants (eight females) specifically for comparing results from the rapid, 2-min MEMR paradigm with the discrete presentation paradigm. Inclusion criteria included normal otoscopic exam, normal tympanograms, and behavioral air conduction thresholds ≤ 15 dB HL at octave frequencies from 0.25 to 8 Hz. All participants reported a negative history of difficulty hearing in quiet and noisy settings, diagnosed or suspected hearing impairment, previous ear surgery (except for pressure equalization tubes in childhood), prolonged exposure to loud noise without wearing hearing protection, dizziness, tinnitus, or use of ototoxic medications. All participants gave informed consent before participating in this study, which was approved by the University of Iowa Institutional Review Board (IRB ID #202202103).

### Equipment

MEMR tests were designed, presented, and responses recorded using a personal computer running custom software, Auditory Research Lab Audio Software [1], developed in MATLAB (The MathWorks, 2024). Acoustic stimuli were delivered, and ear canal pressures were recorded, via a two-channel wideband acoustic probe system (ER-10X; Etymotic Research, Elk Grove Village, IL) connected to a 24-bit sound card (Fireface 802, RME, operating at a 96-kHz sample rate). Each of the two ER-10X channels was associated with an ear tip containing one microphone and two loudspeakers (one input, two outputs). To ensure accurate acoustic measurements across probe insertions, Thevenin-equivalent source calibration was performed using forward pressure level (FPL) calibration, which accounts for standing waves in the ear canal.

### MEMR and SIN Paradigms

All click and broadband noise stimuli were calibrated in individual participant’s ear canals using Thevenin-based methods [20, 35, 40] to deliver flat spectra from 0.1 to 20 kHz. By taking into account source and load impedances, Thévenin-based calibration is able to separate sound pressure level into its forward and reverse components, removing the confounding influence of standing waves in the ear canal. To ensure accurate delivery of stimulus pressure at the eardrum, target levels were specified in dB forward pressure level (dB FPL).

#### Swept Elicitor Paradigm

**Fig. 1:**
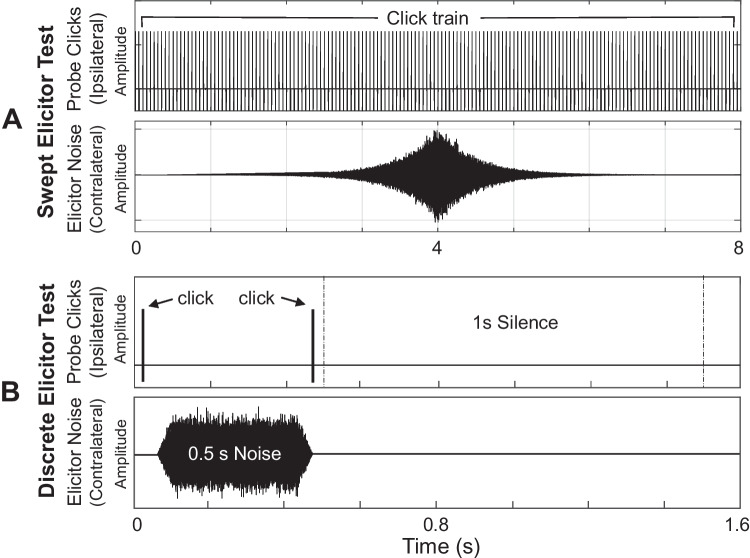
Two measurement paradigms to elicit and record MEMR. **A** Swept elicitor paradigm. MEMR was measured with a train of ipsilateral clicks and a contralateral noise elicitor sweeping from 40 to 110 and back to 40 dB FPL over 8 s. Each sweep was repeated 15 times (2 min total). **B** Discrete elicitor paradigm. MEMR was measured by comparing two clicks separated temporally by a burst of fixed-level contralateral noise. Elicitor noise levels increased from 37.5 to 92.5 dB FPL and decreased back to 35 dB FPL in 5 dB steps, with each level presented 12 times (12 min total)

MEMR was measured using a series of broadband probe clicks (96 dB peak forward pressure level (pFPL) at a rate of 20/s) presented to the ipsilateral (left) ear. These click parameters were selected based on previous wideband MEMR studies and pilot testing. A broadband elicitor noise was presented simultaneously to the contralateral (right) ear to elicit the reflex. The noise elicitor swept upwards in level from 40 to 110 dB FPL in 4 s, then downwards from 110 to 40 dB FPL in 4 s (rate of ± 17.5 dB/s). The starting and ending values of 40 dB FPL were chosen to be just below the ambient noise floor of the ear canal in typical participants. A single sweep spanned a duration of 8 s, during which a total of 160 clicks were presented (Fig. 1A). Each paradigm consisted of 15 sweeps, for a total test time of 2 min. Changes in click ear canal sound pressure as a function of elicitor level had a resolution of one click per 0.875 dB of elicitor change. In other words, the MEMR was measured for every 0.875 dB change in the elicitor level.

#### Discrete Elicitor Paradigm

For comparison purposes, MEMR was measured using broadband probe clicks (96 dB pFPL) presented to the ipsilateral (left) ear. A short burst of broadband noise was presented to the contralateral (right) ear to elicit the reflex. In this paradigm, the probe clicks and elicitor noise were presented sequentially. A single click was presented, followed by a 500-ms noise elicitor, followed by a second click (Fig. 1B). This sequence was repeated 12 times for each elicitor level, with a 1-s interval of silence between the presentation of each sequence. For the main group of 30 participants, elicitor levels increased from 37.5 to 92.5 dB FPL in 5 dB steps. After reaching 92.5 dB FPL, the levels then decreased from 90 dB FPL back to 35 dB FPL in 5 dB steps. For the additional cohort of 8 participants, the elicitor levels were increased to reach a maximum of 110 dB FPL—the same level reached by the swept test. The total duration of the test was 12 min. This paradigm is similar to those described by Mepani et al. [30] and Keefe et al. [21]. Changes in click ear canal sound pressure as a function of elicitor level had a resolution of one click per 5 dB change in the elicitor level.

The major differences between paradigms can be summarized as follows. The swept paradigm tracked reflex changes during a continuous, dynamic presentation of the elicitor, with no silent intervals. The test took 2 min to complete. In contrast, the discrete paradigm measured reflex changes by comparing responses to two clicks, one before and one after a 500-ms elicitor, with a 1-s silent interval between sequences, resulting in a longer duration of 12 min. In addition to reduced test duration, the swept elicitor paradigm provided higher effective measurement resolution (one measurement per 0.875 dB change in elicitor level) compared to the discrete paradigm (one measurement per 5 dB change in elicitor level), potentially offering greater precision in characterizing reflex growth.

#### Speech-In-Noise Testing

Previous research investigating the relationships between MEMR, noise-induced synaptopathy, and SIN performance has employed various SIN tests, including the NU-6 corpus (Auditec) and the QuickSIN™ test (Etymotic Research), among others [17, 30, 32]. In this study, we used a modified version of the QuickSIN (m-QuickSIN) as described by Mepani et al. [30]. The m-QuickSIN test was administered prior to any MEMR measurements, beginning with a practice list consisting of six sentences. Following the practice list, three test lists were presented, each containing six sentences with five key words per sentence. The QuickSIN disc was routed through a GSI 16 audiometer, and the sentences were delivered bilaterally at 70 dB HL through TDH 50P headphones. Background noise consisted of four-talker babble that increased in intensity for each subsequent sentence within a list. Babble presentation levels were 60, 65, 67, 68, 69, and 70 dB HL for the six sentences within a list. The overall score was determined by averaging the number of correctly repeated key words (up to 30 possible per list) from the three test lists.

### Experiment Design

All testing took place in a sound-treated booth. Following the m-QuickSIN, a total of five MEMR tests were administered. Participants completed two swept elicitor tests (Fig. 1A), followed by a single discrete elicitor test (Fig. 1B), followed by two additional swept elicitor tests. The probes were removed from the participants’ ear canals after each test, and a 2-min interval of silence was observed. At the beginning of each test, the probes were reinserted and recalibrated using Thevenin-source methods. Each participant completed the series of five MEMR tests in a single 30-min session.

### MEMR Analysis

**Fig. 2:**
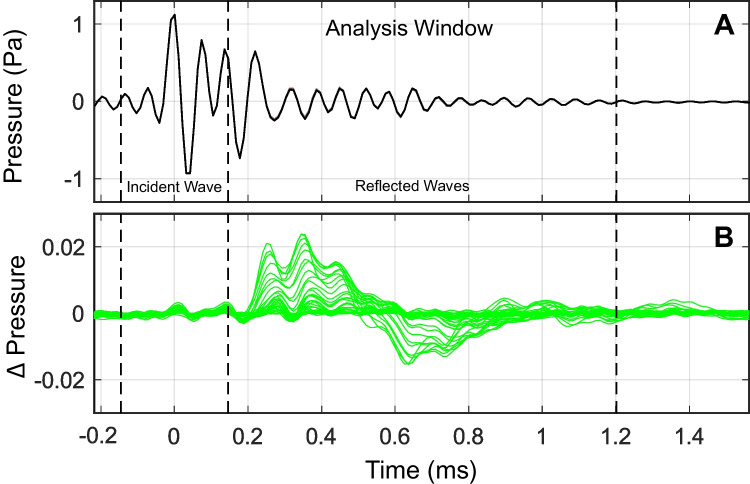
Analysis of ear canal click responses included time windowing to exclude the incident pressure, leaving only the portions that would be influenced by the MEMR. **A** Example of a single click waveform measured in a participant’s ear canal. Time zero was set at the maximum amplitude. Vertical dashes demark regions of the incident wave and multiple internal reflections. **B** Differences in ear canal pressure resulting from MEMR activation (compared to baseline click). No changes are seen in the incident part of the wave, which includes only the forward-going pressure. Changes are seen in the later portions of the click, which contain internal reflections. For visual clarity, only a subset of the total 160 clicks is shown

This section describes the analysis of clicks from the swept elicitor paradigm. Though not described explicitly, to the extent possible the same analysis techniques were applied to clicks obtained from the discrete paradigm. Click stimuli were time-windowed before being transformed using a fast Fourier transform (FFT). The initial incident pressure wave from the probe tip, which contained only forward-going sound energy, was excluded from the analysis. This decision was based on the assumption that MEMR activation would not affect the incident pressure wave but would instead alter the reflected energy, including multiple internal reflections between the probe tip and eardrum (Fig. 2A). A temporal analysis window was determined based on the expected pressure wave travel times in a typical adult ear canal and was empirically validated. For an ear canal length of 1.4 cm and a sound wave speed of 344 m/s, the estimated round-trip travel time is 82 µs. Recorded clicks were windowed using a 1.20-ms flattop window, including 0.146-ms raised cosine onset and offset ramps. The window started at the peak of the recorded click, allowing the onset ramp to remove most of the initial peak energy, leaving unchanged the portions of the waveform dominated by reflected energy. The validity of this window was verified by subtracting the first click from subsequent clicks in the sweep, effectively removing the incident portion and isolating the MEMR-affected energy. As shown in Fig. 2B, MEMR-induced changes were concentrated between 0.2 and 1.2 ms after the click’s peak, with very little change occurring during the incident pressure wave (prior to 0.2 ms).

Windowed clicks were FFT transformed after zero-padding to produce 100-Hz wide analysis bins. Eleven FFT bins centered at frequencies from 500 to 1500 Hz were selected for further analysis. This frequency range was chosen based on the observation that MEMR effects tend to be small above 2 kHz, while environmental and biological noise tends to be large below 500 Hz. Thus, the range from 500 to 1500 Hz should yield the most stable and repeatable results.

**Fig. 3:**
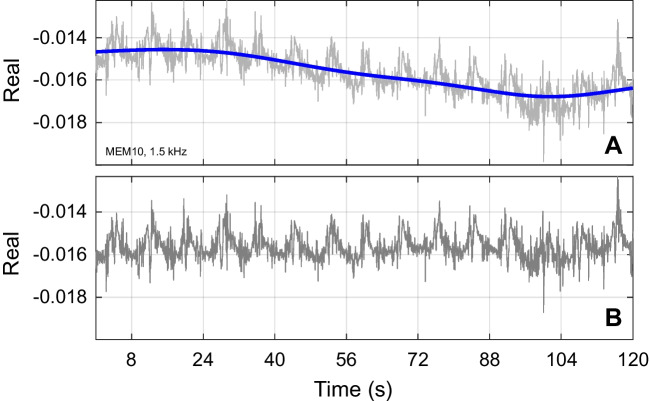
Detrending was used to remove slow changes from ear canal click recordings. This figure shows only the real part of fifteen repetitions concatenated into a single vector for a single participant at one frequency. **A** Gray line shows raw recording. Slow changes are visible over the 2-min recording period. Thicker blue line shows the trend line obtained from a smoothing spline. **B** The same data after subtracting the trend line

Within the 500–1500 Hz frequency range, slow changes were observed across the 15 sweeps (120 s total). The source of these trends is unclear, but one possibility is a slow cochlear modulation involving the central auditory nervous system [2, 24]. Movement of the probe tip as a source of slow changes was ruled out by performing the same analysis on the incident portion of the pressure wave and finding no such changes. Slow changes in each frequency bin were removed using weighted smoothing splines. The 15 sweeps were reshaped into a single column vector, and smoothing splines were applied to the real and imaginary parts separately (Fig. 3A). The smoothed curves obtained from the spline fits were subtracted from the original signal to produce a detrended version. This subtraction removed the slow changes while preserving the higher-frequency components of interest. The detrending splines used a fixed smoothing parameter of 1 × 10⁻⁹, which after subtracting from the raw data was equivalent to highpass filtering with a cutoff frequency of 0.015 Hz. After detrending, the mean value of the smoothing spline (across windows) was added back to the detrended signal to preserve the average value. The real and imaginary parts were then recombined into complex form, and the detrended data were reshaped into their original dimensions for further analysis.

**Fig. 4:**
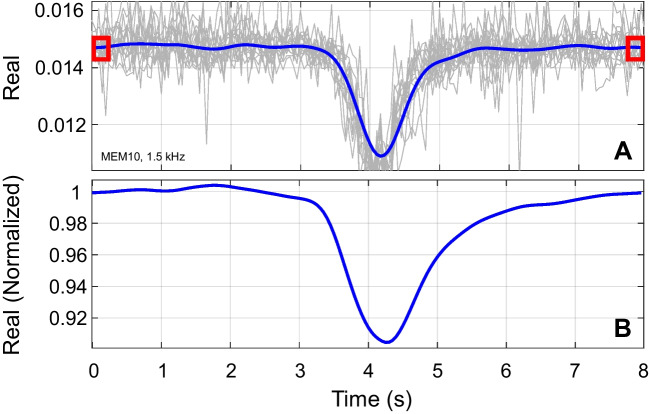
Detrended data were averaged, smoothed, and normalized. The example shown here is from one participant at one frequency, real part only. **A** The 15 sweeps (thin gray lines) were averaged and smoothed (thicker blue line). The first three and last three click responses (areas within the red rectangles) were averaged to establish a baseline. **B** Responses after normalization by baseline

Following detrending, the data in each frequency bin were averaged and then again smoothed with weighted smoothing splines (done separately for the real and imaginary parts). These splines had less smoothing than the splines used for detrending (above), with the smoothing parameter chosen to capture the essential rise and fall of the MEMR response while reducing the higher frequency noise (Fig. 4A). All of these splines used a fixed smoothing parameter of 0.001, which was equivalent to lowpass filtering with a cutoff frequency of 0.46 Hz. Weighting was used to down-weight or remove the influence of any noisy portions of the recording, providing artifact control. In summary, detrending removed slow changes less than approximately 0.015 Hz, and smoothing removed fast changes with frequencies greater than approximately 0.46 Hz. The slow changes that were removed by detrending may be part of the MEMR, but such changes have not traditionally been considered and were not a focus of the current study. The fast changes that were removed were assumed to be noise.

Finally, to assess relative MEMR-induced changes, the averaged and smoothed data were normalized to baseline values. Baseline in each frequency bin was computed as the average of the first three and last three responses (red rectangles in Fig. 4A). The entire smoothed signal was divided by this baseline, resulting in a normalized signal where each point represents the MEMR-induced complex change in response to the elicitor. Figure 4B shows the normalized response for one recording (real part only).

#### Complex Analysis and Total Change

**Fig. 5:**
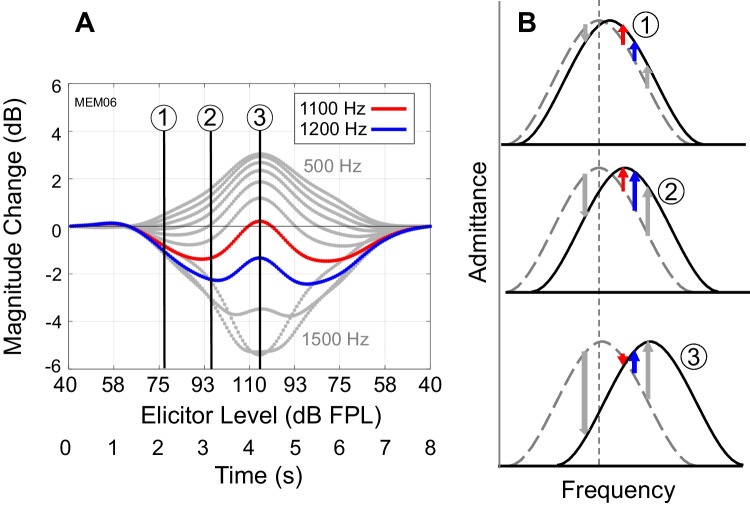
Patterns of change at different frequencies (**A**) can be explained by a simple model (**B**) of MEMR-induced stiffening of the middle ear and shifting of the resonant frequency. **A** Relative magnitude changes in ear canal pressure as a function of frequency, elicitor level, and time. Example data from one participant. Each gray curve shows the response at one frequency (in 100 Hz steps between 500 and 1500 Hz). Two frequencies exhibiting non-monotonic growth are shown in red and blue. Three time points in the LGF are marked with numbers 1, 2, and 3, and applied to a conceptual model of MEMR in (**B**). **B** A simple bandpass model of middle ear admittance predicts the frequency-specific growth patterns observed. Drawings labeled 1, 2, and 3 correspond to the time points and elicitor levels indicated in Panel A. In all three cases, the dashed gray curve indicates baseline admittance (no MEMR), with the resonance frequency shown by the dashed vertical line. As elicitor level increases, the MEMR exerts a stronger stapedius muscle contraction, increasing stiffness and shifting the resonance to higher frequencies, as shown by solid black curves. For frequencies either below or well above baseline resonance (gray vertical arrows), growth is monotonic. For frequencies slightly above baseline resonance (red and blue vertical arrows), growth is non-monotonic and can sometimes cross zero and change directions (red arrow, time point 3)

Understanding the combined magnitude and phase changes in ear canal SPL is critical to our approach for averaging MEMR-induced effects across different frequencies. These changes provide insights into how MEMR activation alters middle ear mechanics, including stiffness and impedance [48]. Initially, MEMR LGF changes were considered in terms of magnitude change only. As shown in Fig. 5A, at frequencies below middle ear resonance (at 500 Hz for example), activation of the MEMR led to an increase in ear canal SPL, consistent with stiffening of the ossicular chain, an upward shift in middle ear resonant frequency and increased middle ear impedance, and thus reflection of sound at the ear drum at this frequency. Conversely, at frequencies above middle ear resonance (at 1500 Hz for example), activation of the MEMR led to a decrease in ear canal SPL, also consistent with an upward shift in middle ear resonant frequency and reduced middle ear impedances and thus increased transmission at these frequencies. However, at frequencies near middle ear resonance (typically between 800 and 1200 Hz), responses showed non-monotonic magnitude growth patterns in response to strictly increasing (i.e., 40–110 dB FPL) or decreasing (i.e., 110–40 dB FPL) elicitor levels (Fig. 5A, red and blue lines). Such non-monotonic magnitude changes are consistent with a shifting middle ear resonance due to increasing MEMR-induced stiffness, as illustrated in Fig. 5B. This raised a potential problem, in that some frequencies exhibited monotonic magnitude changes, while other frequencies, under the influence of the same reflex, showed non-monotonic magnitude changes. Including magnitude changes from frequencies with non-monotonic responses could alter the slope of the changes as well as underestimate the strength of the reflex. However, such frequencies, which are near middle ear resonance, have the potential to be sensitive to MEMR-induced changes, making it undesirable to simply remove them from subsequent analyses. Our solution to this problem is to include phase information in our MEMR measures.

**Fig. 6:**
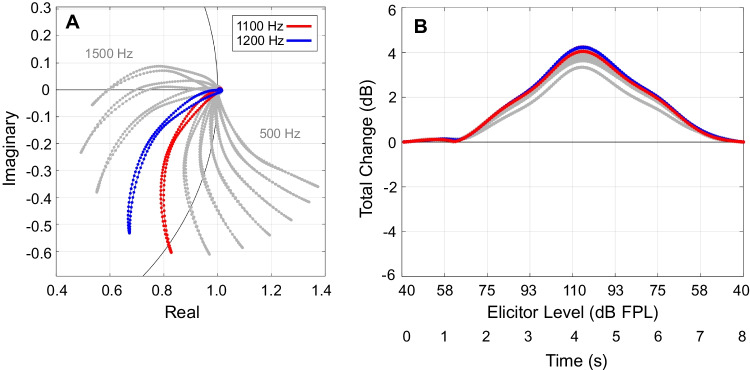
Changes in both magnitude and phase were combined to create total change LGFs. Each gray curve shows the response at one frequency, and red and blue lines denote responses at 1100 Hz and 1200 Hz, respectively. **A** Complex relative changes in ear canal pressure as a function of elicitor level (same participant as in Fig. 5). A portion of the unit circle is shown in black. Portions of the curves falling on the unit circle have no magnitude change. Portions of the curves falling inside the circle show a magnitude decrease, while portions falling outside the circle show a magnitude increase. Portions falling at zero on the y-axis have no phase change. Portions with positive y-axis values have a phase lead, while portions with negative y-axis values have a phase lag. **B** Ear canal pressure as a function of elicitor level expressed as total change

Joint magnitude and phase changes can be visualized by plotting MEMR-induced change in the complex plane (Fig. 6A). Each of the “pinwheel arm” traces represents measurements at one frequency. Each trace moves from baseline (no change) at the point 1 + 0*i* on the complex plane to some extrema as the elicitor increases, and then back to baseline as the elicitor decreases. Portions of the trace where the movement away from and back to baseline do not overlap are indicative of MEMR hysteresis. Examination of Fig. 6A shows that curvature in the complex trace with changes in MEMR-induced stiffness is related to non-monotonic magnitude growth. Specifically, some frequencies (1100 and 1200 Hz in Fig. 6A) initially move from the starting point towards the origin on the unit circle, but the curvature then moves them back towards the unit circle. Such curved trajectories have been reported previously (e.g., Lutman and Martin [27], Figs. 4 and 8).

If the lengths of the traces are computed via complex integration, the responses of all frequencies show similar growth patterns, conducive to subsequent averaging to obtain a single mean measure of change across frequency. The lengths of each trace can be found by computing $\int k\sqrt{{\left(dx/dt\right)}^{2}+{\left(dy/dt\right)}^{2}} dt$, where $x$ and $y$ are the real and imaginary parts of the complex change vector, respectively, $t$ is the corresponding time vector, and $k$ is assigned a value of + 1 when the stiffness is increasing and −1 when the stiffness is decreasing. The changing sign of $k$ results in an integration that is monotonically increasing to the point of maximum stiffness change, and thereafter monotonically decreasing. A nearly identical result can be obtained more simply by subtracting 1 from the complex change vector and then computing magnitude: $\sqrt{{\left(x-1\right)}^{2}+{y}^{2}}$. This operation shifts the baseline of the complex change vector to the origin before computing magnitude, so that both magnitude and phase are combined into a single, positive, real number. We therefore refer to this measure as “total change.” Total change has been referred to previously in studies of the medial olivocochlear reflex [4, 16, 31].^1^ Figure 6B shows an example of MEMR changes expressed as total change. Across the frequency range considered in this study (500–1500 Hz), total change was monotonic across strictly increasing or strictly decreasing ranges of elicitor level, and less variable across frequency than magnitude alone (compare Fig. 5A to Fig. 6B as a representative example).

## Results

### Swept Elicitor Paradigm

**Fig. 7:**
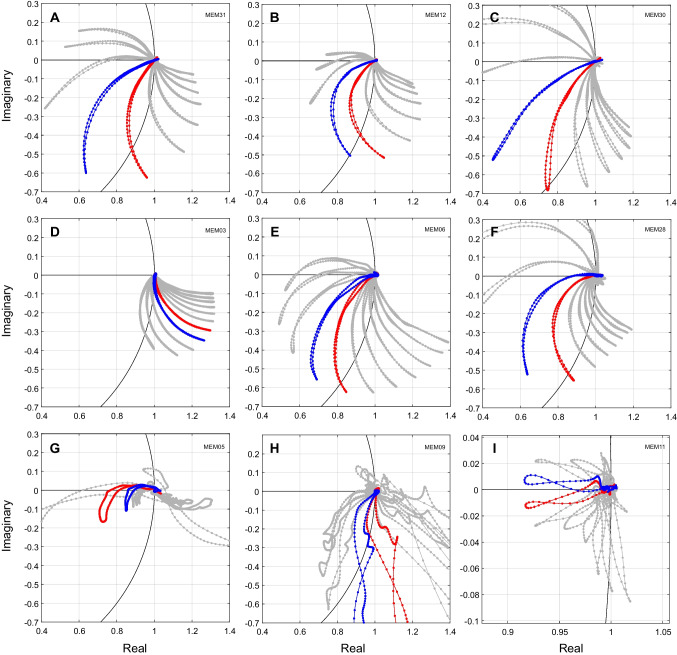
Joint magnitude and phase plots showing the variability of relative MEMR change across participants. Each panel (**A-I**) shows data from one participant. Formatting is the same as Fig. 6A. Each gray curve shows the response at one frequency. A portion of the unit circle is shown in black. Red and blue lines indicate 1100 and 1200 Hz, respectively

**Fig. 8:**
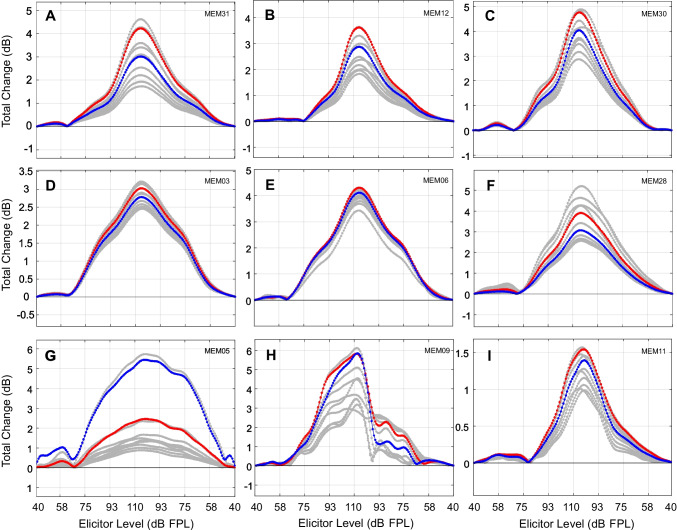
Total change LGF plots showing the variability of relative MEMR change across participants. Total change in MEMR activation across frequency for the same participants (**A-I**) shown in Fig. 7. Formatting is the same as Figs. 6B and 7. Each line shows data from one frequency bin. Red and blue lines show responses at 1100 Hz and 1200 Hz, respectively

To illustrate the variability observed across participants, Fig. 7 shows joint magnitude and phase change plots from nine participants. The top six panels show smooth responses consistent with good signal-to-noise ratio. The bottom three panels show noisier responses. Out of 120 swept MEMR tests (30 participants × 4 repeats), 100 tests (83.3%) showed results similar to the top six panels, and 20 tests (16.7%) showed noisier results similar to the bottom three panels. Note that the locations in the complex plane of the 1100 Hz and 1200 Hz traces (red and blue lines, respectively, in Fig. 7) varied in location for some participants (e.g., panels D and I). This is consistent with individual differences in middle ear resonance and supports the utility of calculating total change and averaging across frequencies, rather than choosing different frequencies for each participant in an attempt to avoid frequencies near resonance. This is particularly the case since, as seen in Fig. 7, the largest excursions (potentially with the highest sensitivity to MEMR change) are often those near resonance. Figure 8 shows the same participants’ data expressed as total change. Total change always showed more consistent changes and was less variable across frequency than magnitude alone.

**Fig. 9:**
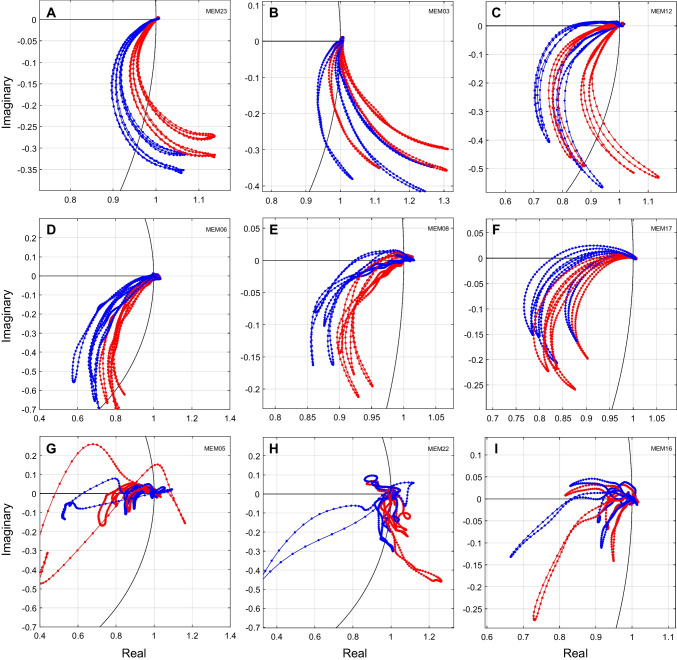
Joint magnitude and phase data at 1100 and 1200 Hz, which frequently have non-monotonic magnitude changes (e.g., Fig. 5A). Each panel (**A-I**) shows four repeated measurements from one participant, illustrating retest variability. Red and blue lines, respectively, indicate 1100 and 1200 Hz, and the black curve shows a portion of the unit circle. Formatting is the same as Fig. 6A

To illustrate the consistency across repeated measurements within participants, Fig. 9 shows joint magnitude and phase change plots from nine participants that differ from those shown in Figs. 7 and 8, with four repeated measurements for each participant. For visual clarity, only 1100 and 1200 Hz are shown. Qualitatively, the variability across participants appears greater than the variability across repeated measurements within individuals. Quantitative measures are reported in the next section.

#### Characterizing Swept Elicitor LGF Measures

**Fig. 10:**
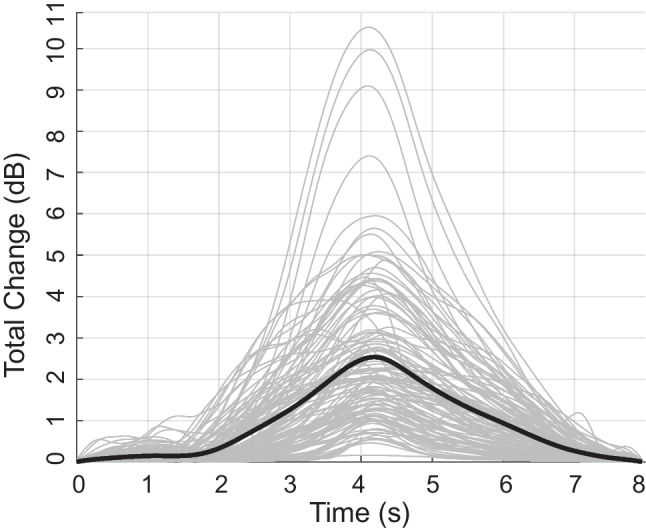
Group data showing MEMR LGFs for the swept elicitor paradigm. Thin gray lines show individual measurements, and the thick black line indicates the median change across all 120 measurements

**Fig. 11:**
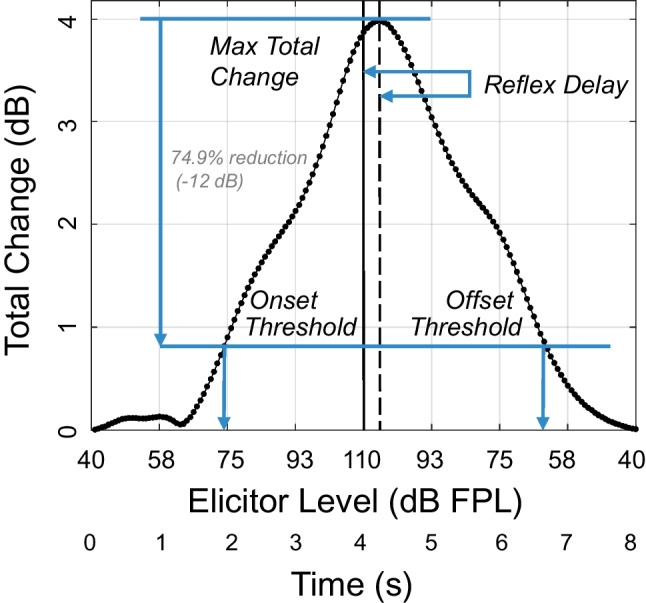
LGFs were characterized by five key measures: maximum total change, reflex delay, onset and offset thresholds, and hysteresis (defined as the difference between onset and offset thresholds)

LGFs showing MEMR-induced change in ear canal pressure were computed as the mean total change across the 11 analysis frequencies. Figure 10 shows the LGFs obtained for all 120 swept MEMR tests. All participants in this study showed LGFs consistent with MEMR activation. Despite all participants having normal hearing, considerable variability in the size of changes was observed across participants. As shown in Fig. 11, averaged LGFs were summarized by five key measures: (1) *Maximum total change* was the largest change that occurred in the LGF; (2) *Reflex delay* was the difference between the time at which the maximum elicitor level was presented (4 s) and the time at which the maximum total change was observed. This value was typically in the 100–200 ms range, consistent with reported MEMR reflex delays [33]. (3, 4) *Onset and offset thresholds* were defined as the elicitor level producing a response amplitude with a 74.9% reduction (−12 dB) relative to the maximum total change (after shifting the function to account for reflex delay). Note that total change was expressed in dB relative to baseline, while thresholds were defined as a dB reduction relative to maximum total change. Because the noise floor itself is a variable estimate, and because we found that noise floors sometimes fluctuated across repeated tests, we found this method of calculating thresholds gave the most dependable response across repeated tests. (5) *Hysteresis* (in dB) was quantified as offset threshold minus onset threshold.

One hundred twenty swept elicitor measurements were obtained from 30 participants, all of whom exhibited LGFs consistent with a MEMR response. Across all measurements, the median values for the five key measures were 2.18 dB maximum total change, 210 ms reflex delay, 87.18 dB FPL onset threshold, 72.27 dB FPL offset threshold, and −14.91 dB hysteresis. (The hysteresis result means that thresholds for decreasing elicitors were almost 15 dB lower than thresholds for increasing elicitor levels.)

**Fig. 12:**
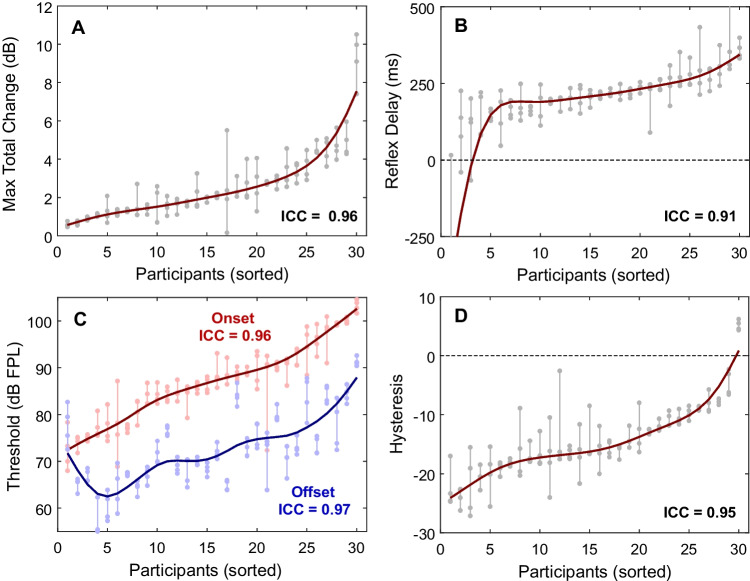
Retest repeatability was excellent for measures used to characterize the LGFs. In each panel, participants were ordered according to the mean of their repeated measurements for (**A**) total change, **B** reflex delay, **C** onset and offset thresholds, and (**D**) hysteresis. Gray dots connected by vertical gray lines represent the data from each participant. Thick lines help show the distribution of values across participants. The ICC values demonstrate excellent repeatability and reliability for all measures

The repeatability of the swept elicitor measurement for the five LGF-derived measures was assessed using intraclass correlation coefficients (ICC, type “C-k,” McGraw and Wong [29]). The ICC values indicated excellent reliability for maximum total change (0.96), reflex delay (0.91), onset threshold (0.96), offset threshold (0.97), and hysteresis (0.95). Figure 12 shows the repeatability of each measure across four repeated tests for each participant, along with their respective ICC values. These findings suggest that total change LGFs in the swept elicitor paradigm provide highly consistent results across repeated tests within individuals.

#### Comparison of LGFs Evoked from Swept and Discrete Elicitors

**Fig. 13:**
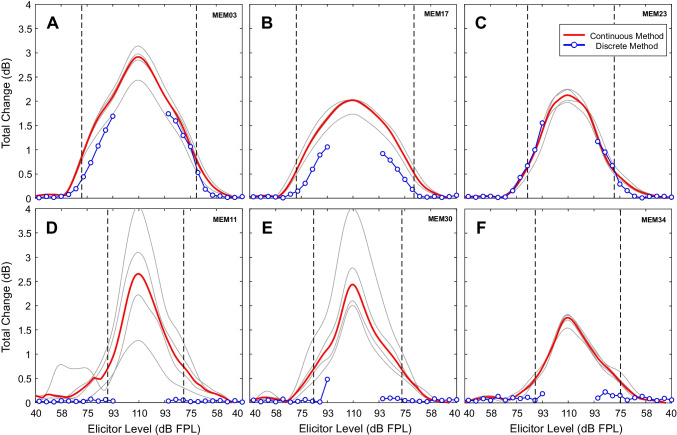
MEMR LGFs evoked from swept elicitors had larger amplitudes than those evoked from discrete elicitors at elicitor levels where both measurements were available. Thus, the threshold of MEMR evoked from swept elicitors was lower. Each panel (**A-F**) shows swept (solid red) and discrete LGFs (blue circles) from one participant. The break in the discrete measurements near the center of each panel results from the highest elicitors not being tested in the discrete paradigm. Swept results are shown by solid lines with no markers. The four repeated swept measurements are shown by thin gray lines, and the median is shown by a thicker red line. Vertical dashed lines show onset and offset thresholds for the swept paradigm. The swept paradigm provided a measure of reflex delay, but the discrete paradigm did not. To aid visual comparisons, the swept LGFs in this figure have been appropriately shifted along the x-axis by subtracting the measured reflex delay from the time vector. Top row: Two-thirds of participants had results similar to these examples. Bottom row: One-third of participants did not show results consistent with a present MEMR when assessed with the discrete method, though their swept results clearly showed the presence of MEMR

As described in Fig. 10, all 30 participants in the main study had measurable MEMR evoked with the swept elicitor paradigm. For these participants, the discrete MEMR paradigm did not include elicitor levels greater than 92.5 dB FPL due to technical oversight. For this main study, comparisons of measurements from swept and discrete elicitor paradigms were made at lower sound levels. Only two-thirds of participants (20/30) had detectable MEMR with the discrete paradigm. Representative examples of MEMRs that were present in the discrete paradigm are shown in the top row of Fig. 13, and examples of representative participants that did not show a MEMR in the discrete paradigm are shown in the bottom row of Fig. 13. For the participants with present MEMR in both paradigms, LGFs obtained with the discrete paradigm were similar in shape to those obtained using the swept paradigm (Fig. 13, top row), albeit the overall LGF amplitudes tended to be lower in the discrete paradigm.

Additional MEMR data were collected from eight participants, who were not participants in the main study. For these additional measurements, the swept and discrete paradigms had equivalent elicitor levels (i.e., up to 110 dB FPL for both paradigms). The swept results from these additional participants produced curves that were indistinguishable from the results of the 30 participants shown in Fig. 10. All eight of these additional participants had detectable MEMR with both paradigms.

**Fig. 14:**
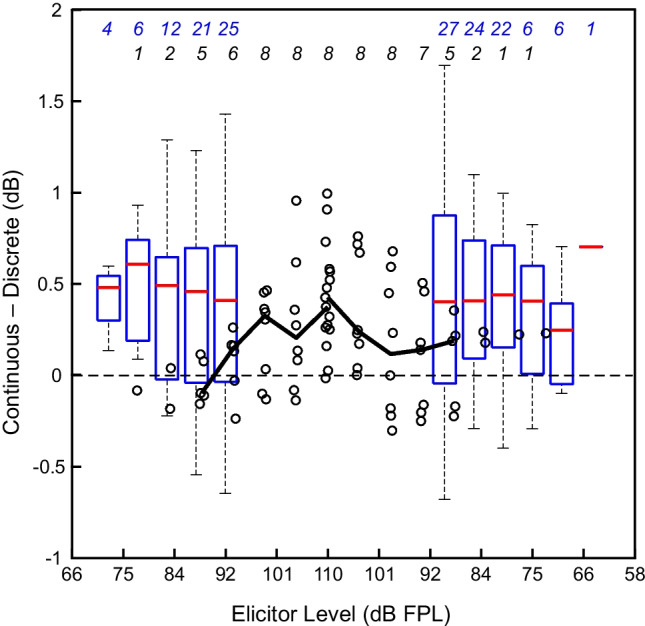
Total change was larger for MEMR evoked by the swept paradigm compared to the discrete paradigm. Values from the discrete paradigm were subtracted from corresponding swept values (median of the four repeats). Data points used for comparison were those at discrete elicitor levels greater than the swept onset and offset thresholds that also showed MEMR on the discrete paradigm. Data from the 30 participants of the main study are shown with box and whisker plots, and data from the additional 8 participants are shown with open black circles. Thick black lines connect median differences for which data from at least four participants were available. The number of participants contributing to the box and whisker plots at each level is shown along the top of the figure, with the first row of numbers in blue referring to the 30 participants of the main study and the second row referring to the additional 8 participants

To ensure a meaningful quantitative comparison of continuous and discrete LGFs, differences were considered only at discrete elicitor levels which were greater than or equal to the continuous thresholds of each participant. These comparison points are those shown by the blue circles that fall inside the pairs of vertical dashed lines in each panel of Fig. 13. Figure 14 shows the dB differences in total change between LGFs obtained with the two methods. Box and whisker plots show the differences for the 30 participants in the main study, and open black circles show the results from the additional 8 participants. Taken together, the data suggest that differences were similar across elicitor levels, with the continuous LGFs from the 30 participants being 0.41 dB larger on average (SD = 0.62 dB). The continuous LGFs from the additional 8 participants were 0.22 dB larger on average (SD = 0.31 dB).

### Correlations Between MEMR and Speech in Noise Performance

**Fig. 15:**
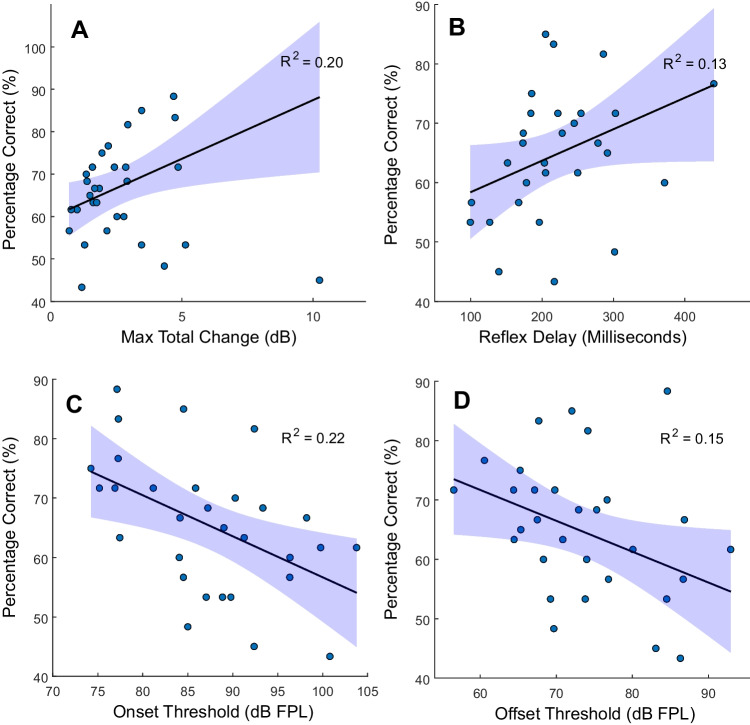
Scatter plots illustrating the relationship between m-QuickSIN scores and (**A**) maximum total change, **B** reflex delay, **C** onset thresholds, and (**D**) offset thresholds. Shaded areas indicate 95% confidence intervals

Robust linear regression was used to examine the relationships between m-QuickSIN scores and swept LGF MEMR measures. Bisquares re-weighting was used to reduce the influence of data points far from the mean. The coefficient of determination, ${R}^{2}$, was calculated to assess how much of the variation in the dependent variable was explained by the predictor variable. To evaluate the significance of the regression slope, confidence intervals (CIs) for the slope coefficient were examined. If the CIs excluded zero, this suggested a relationship between the predictor and the m-QuickSIN scores. Results are shown in Fig. 15. Hysteresis was not included in this figure, because its regression CI encompassed zero (${R}^{2}=0.025$).

Results in Fig. 15 suggest a relationship between SIN scores and MEMR measures, particularly maximum total change and onset threshold (Fig. 15A and B). Lower onset and offset thresholds were associated with better SIN performance, while maximum total change was positively correlated, suggesting that a more robust MEMR may be linked to better SIN performance. However, we view these initial results cautiously, as additional studies are needed to understand how MEMRs evoked with a swept-elicitor can be used to objectively assess the common complaint of real-world difficulty hearing in noise.

## Discussion

### Comparison of Swept- and Discrete-Elicitor MEMR Paradigms

With excellent repeatability and efficient stimulus presentation, our new MEMR paradigm with a continuously swept elicitor may be well suited for both clinical and research applications. In general, the swept elicitor paradigm showed LGFs that were qualitatively similar to those measured with a more traditional discrete paradigm. Quantitatively, the swept elicitor paradigm yielded total change values that were slightly larger than those obtained by the discrete paradigm (between 0.2 and 0.4 dB on average). Reflex delay could not be estimated with the discrete paradigm because the two probe clicks were temporally distant from one another. Hysteresis could be observed using both the swept and discrete paradigms, though it was unclear whether methodological differences affected this measure. In a few cases (e.g., Fig. 13A), there appeared to be differences in hysteresis measured by the two methods. However, in many cases, there was apparently no difference (e.g., Fig. 13B, C). These results suggest that the swept paradigm may increase the amount of potentially useful diagnostic information without sacrificing essential features obtained with traditional discrete paradigms.

The swept elicitor test was faster than the discrete comparison test we used (2 min versus 12 min). One reason was the 1-s silent period after each discrete presentation, ostensibly to allow the MEMR to return to baseline between each presentation. However, it was unclear whether the silent period was advantageous, given the discrete responses were smaller than the swept responses. A sure disadvantage of including a silent period is the increased test time. We note that traditional MEMR reflex tests used in clinical audiology likely do not allow enough time for the reflex to return to baseline.

It has been shown that the magnitude of MEMR may exhibit increases over time when repeatedly stimulated [2]. In the current study, gradual changes were evident, with most participants showing an increasing trend in response magnitude over the course of each 2-min swept-elicitor test (see Fig. 3). Rather than presenting a silent period to allow repeated returns to baseline, our approach was to present continuously and detrend the results as part of the analysis. Even with detrending, results from the swept paradigm showed clear evidence of hysteresis, in that MEMR responses were different for ascending compared to descending elicitors. Hysteresis measures in the current study showed that the MEMR threshold was nearly 15 dB lower for decreasing elicitor levels compared to increasing levels. These results are consistent with the findings reported by Baricevich et al. [2], who demonstrated similar effects using a series of ascending and descending elicitor levels. In their study, hysteresis was consistently observed beginning with the first descending series following the initial ascending series, and the hysteresis persisted across subsequent ascending–descending series. Future research may determine how this effect varies with hearing impairment or disease and determine the usefulness of hysteresis for differential diagnosis.

The MEMR is a dynamic nonlinear process, with activation and relaxation having different time constants [5, 11]. The hysteresis observed in our swept-elicitor paradigm may be influenced by aspects of the neural pathway involved in the reflex. It may also be strongly influenced by the nonlinear dynamics of muscle contraction and relaxation. In this study, we did not attempt to separate neural and muscle fiber contributions. Rather, we interpreted hysteresis broadly as a functional characteristic of the MEMR system reflecting its dependence on increasing versus decreasing elicitor level. The connection between MEMR hysteresis and hearing health has not been well studied, and future studies may provide insight into this relationship.

### Comparison to Standard Clinical Probe Frequency

Although lower frequencies exhibit larger MEMR-induced magnitude changes, they are also subject to increased biological noise floor below 500 Hz, leading to poorer SNR and greater variability. Conversely, MEMR magnitudes diminish at higher frequencies, reducing detectability. To balance these trade-offs, we selected the 500–1500 Hz range, where the reflex-induced change remains well measurable, and SNR is sufficiently high. We hypothesized that averaging changes across this range, rather than relying on a single frequency, would enhance repeatability by reducing frequency-specific noise contributions. We evaluated test–retest reliability (via ICC) across the probe frequency range and compared it with ICCs computed using only a single probe frequency of 200 Hz. As hypothesized, use of a single low-frequency bin of 200 Hz (similar to the clinical standard of a 226-Hz tone) yielded significantly more variable results. For the 200 Hz probe, the ICC values were 0.93, 0.36, 0.76, 0.85, and 0.63 for total change, reflex delay, onset threshold, offset threshold, and hysteresis, respectively. In contrast, use of the averaged response from 500 to 1500 Hz yielded ICC values of 0.96, 0.91, 0.96, 0.97, and 0.95, respectively. These findings support previous work showing that higher frequency, wider bandwidth probes have some advantages for quantifying MEMR [12, 38].

### Limitations and Challenges

A limitation of the swept paradigm described in this paper is that the use of elicitors is restricted to the contralateral ear. The paradigm could be adapted for ipsilateral and bilateral tests by introducing short (5–10 ms) temporal notches in the elicitor noise at locations surrounding the clicks. This approach is expected to slightly reduce the effective elicitor level, but this can be compensated for by adjusting the overall levels. Initial modifications in our lab showed that the notched elicitor noise produced essentially the same results as the current unnotched version, once overall levels were equated.

Another limitation of the swept paradigm described in this paper is that short clicks with broad bandwidths and flat magnitude spectra are required for efficient time windowing. Our study used extended bandwidth probes and careful calibration, but some loudspeakers may not be able to achieve short enough clicks to make time windowing practical. Time windowing was done to exclude the incident portions of the recorded click, focusing the analysis on only the reflected portion of the waveform. We compared results obtained with and without this temporal windowing. Excluding the incident portion resulted in observed effects that were larger by 1.27 dB, on average. While this temporal windowing helps achieve the largest effects, in situations where such windowing is not feasible, failure to remove the incident portion will likely not have large negative effects on test performance.

An important question, particularly for potential clinical utility, is whether the amount of time to complete our new test could be further shortened with only minor decrements in test performance. We briefly addressed this question by reducing the number of sweeps from 15 to 4, and then to 1 (i.e., test time was reduced from 2 min to 32 s, and then to 8 s). These reductions had minimal impact on ICC values for detecting maximum change, but larger decreases in ICCs for onset thresholds (from 0.97 to 0.72, and then to 0.60). These results were expected, because the maximum change is calculated from portions of the response with high SNR, while thresholds are computed from areas with lower SNR. If maximum change is the measure of interest, then the paradigm can be substantially shortened. If, however, thresholds or hysteresis are the measure of interest, the paradigm in its current form likely cannot be substantially shortened without negatively impacting repeatability.

The ICCs reported in this study were obtained using repeated tests obtained within a single visit to the lab. Therefore, the majority of the observed variability was likely from probe replacement. The repeatability of MEMR over longer timelines, spanning from days to months, has been investigated in some of the recent studies. Thresholds appear to vary by up to 10 dB, whereas reflex magnitudes vary by less than approximately 1 dB. Bramhall et al. [8] tracked contralateral wideband MEMR thresholds for up to 5 months and found a mean absolute test–retest difference of nearly 10 dB (95% CI 8–13 dB). Feeney et al. [15] reported a nearly identical spread: 10.2 dB for ipsilateral wideband thresholds in normally hearing adults. Schairer et al. [39] likewise observed a 10–12 dB variability in threshold. In contrast, for MEMR magnitude, the Bramhall et al. study reported the median between-session change in reflex magnitude was only 0.23 dB, roughly one-fifth of the median magnitude response itself (1.14 dB). Future investigations may establish the repeatability of our new paradigm over longer periods of time and across multiple lab visits, which is essential for using this test to describe the time course of disease and efficacy of treatments.

This study included approximately equal numbers of female and male participants; however, it considered the results as a whole and did not consider possible differential effects of sex on MEMR. At the present time, effects of sex on MEMR are not considered in clinical audiology. Future work should be designed to consider this variable.

### Relationship of MEMR Measures and Speech in Noise Performance

Some listeners have difficulty understanding speech in noisy environments despite having normal audiometric thresholds (sometimes referred to as “hidden hearing loss”). This condition may arise in part from the loss of synapses between inner hair cells and afferent auditory nerve fibers, particularly high-threshold fibers that are involved with encoding suprathreshold speech sounds (e.g., [23]). The synaptopathy associated with hidden hearing loss is believed to be correlated with a deficient or weakened MEMR [30, 41]. We examined correlations between participants’ performance on the m-QuickSIN and five measures derived from MEMR LGFs, including onset threshold, offset threshold, and total change. We observed negative correlations between MEMR thresholds and m-QuickSIN scores, as well as positive correlations between total change and m-QuickSIN performance.

MEMR threshold correlations with SIN performance have shown mixed results across studies. Mepani et al. [30] observed no significant relationship between MEMR thresholds and m-QuickSIN scores, contrasting with our findings. However, they reported significant negative correlations between MEMR threshold and NU-6 word recognition scores when speech-shaped noise at 0 dB SNR was added, or when words were time-compressed (45% or 65%) with reverberation. Correlations between MEMR magnitude and SIN performance have generally shown weaker associations. Maximum activation measures in the present study are similar to what Mepani et al. [30] referred to as “MEMR strength,” which they defined as the summed absolute gain values across specific frequency bands. In their results, MEMR strength correlated significantly with SIN performance only for the 45% time-compressed condition. Similarly, Shehorn et al. [41] measured MEMR magnitude in response to broadband noise activator levels of 74, 89, and 104 dBA using the Maryland CNC test [10] and found a significant positive correlation only at the highest activator level (104 dBA). The current study likewise identified a significant correlation at a relatively high level (110 dB pFPL), though direct comparisons of stimulus levels across studies are limited by methodological differences (e.g., flat versus A weighting, continuously sweeping versus steady levels). Overall, results from our study are generally consistent with some results reported by others. While the goal of our current work was to develop a new measure using a homogeneous group of young adults with normal hearing, future research can assess the correlation between MEMR and SIN scores in populations where this relationship is expected to be more pronounced, individuals with hearing loss, tinnitus, and/or those who report difficulty listening in real-world noisy environments.

## Conclusions

Our report describes a novel paradigm for evoking and measuring MEMR, in which a train of broadband clicks act as probes, while broadband noise elicitor continuously sweeps in level. Total change, a new measure of MEMR activation, was introduced to address non-monotonic magnitude growth observed at frequencies near middle ear resonance. Total change combines magnitude and phase changes into a single value, simplifying the averaging of LGFs across frequency. MEMR LGFs were characterized with measures of maximum total change, reflex delay, onset threshold, offset threshold, and hysteresis. These measures demonstrated excellent repeatability. A key strength of our test is its ability to produce repeatable, low-variability MEMR measurements across sessions and individuals, which may improve comparability across studies and support future clinical applications. Comparison of these measures with scores on a SIN test provided encouraging suggestions of a relationship between MEMR measures and SIN performance.

## Data Availability

The data generated during and/or analyzed during the current study are available from the corresponding author on reasonable request.

## Footnotes

1 For some original waveform $a$ and a second waveform $b$, total change is computed as:

1 $$\begin{array}{c}A=F\left(a\right),B=F(b)\\\Delta=B/A\\\nabla=\left|\Delta-1\right|\end{array}$$

where $F\left(\cdot \right)$ indicates the discrete Fourier transform, $\Delta$ is a vector of relative complex changes, and $\nabla$ is the total change vector. For two sinusoidal waveforms $a$ and $b$, of identical frequency but possibly different magnitudes and/or phases, the energy in $A$ and $B$ is concentrated in a single frequency bin, with negligible energy elsewhere. In this case, total change in that same frequency bin *f* is equal to the relative RMS change, which can easily be calculated in either in the time or frequency domains:

2 $$\nabla_f=\sqrt{\sum{(b-a)}^2}/\sqrt{\sum a^2}=\sqrt{\sum{\vert B_i-A_i\vert}^2}/\sqrt{\sum{\vert A_i\vert}^2}$$

For two waveforms $a$ and $b$, containing multiple frequencies, total change can be made equal to the relative RMS change by computing the energy-weighted mean of $\nabla$ across frequency:

3 $$\sqrt{\sum{(\nabla\left|A\right|)}^2/\sum{\vert A\vert}^2}=\sqrt{\sum{\vert B_i-A_i\vert}^2}/\sqrt{\sum{\vert A_i\vert}^2}$$

In this case, each frequency bin is weighted by the power in original signal. When considering ear canal pressures measured at the entrance of the canal, and assuming the incident part of the waveform is windowed out, the frequencies with the largest power are generally expected to be low frequencies, where middle ear impedance is larger, resulting in greater reflected energy. However, the frequencies with the most sensitivity to MEMR change are closer to middle ear resonance (Fig. 8). Combining total change across frequency by computing the energy-weighted mean is therefore less desirable, because it gives more weight to frequencies that are less sensitive to change. In this paper, we therefore combined total change across frequency by computing the standard, non-weighted mean of $\nabla$.

## Code Availability

Code used to analyze the data is available on the corresponding author’s GitHub repository at https://github.com/myKungFu/MEMR.

## Declarations

### Use of AI

No traditional or generative AI technologies were used for any aspect of this project.
